# Supplementary material for: SyNDI: synchronous network data integration framework
Source: BMC Bioinformatics. 2018 Nov 6;19:403. doi: 10.1186/s12859-018-2426-5 (PMC6219086; doi:10.1186/s12859-018-2426-5)
Supplement: Supplementary file 5 — Files of the example presented in Additional file 4. This additional file is a zip package that contains all files used in Additional file 4. Detailed descriptions of each of these files are presented in a README file included in this zip package. (GZ 30411 kb) [file 12859_2018_2426_MOESM5_ESM.gz › SyNDITools/Meme2Fimo/meme_4.9.0_4.tar.gz!/meme_4.9.0/doc/tamo2meme.html]

tamo2meme


# tamo2meme

**Usage:** `tamo2meme [options] <matrix file>`

**Description:**

Convert a TAMO file into a MEME version 4 formatted file
suitable for use with MAST and other MEME Suite programs.

**Input:**

A TAMO file describes one or more motifs in terms of a Log-odds matrix
and a set of sites that were used to make the motif. The script reads
the sites and converts them into a position specific probability matrix.

A probability matrix and optionaly a log-odds matrix is output
for each motif in the tamo file. The probability matrix is computed
using pseudo-counts consisting of the background frequency
(see -bg, below) multiplied by the total pseudocounts
(see -pseudo, below). The log-odds matrix uses the background
frequencies in the denominator and is log base 2.

**Options:**

-skip <TAMO ID>
:   skip this ID (may be repeated)

-numbers
:   use numbers instead of strings as motif names

-bg <background file>
:   file with background frequencies of letters; default: uniform background

-pseudo <total pseudocounts>
:   add <total pseudocounts> times letter background to each frequency; default: 0

-logodds
:   print log-odds matrix, too; default: print frequency matrix only

-url <website>
:   website for the motif; The tamo ID is substituted for MOTIF\_NAME; default: no url

**Output:**

Writes MEME format to standard output.

**Sample Input:**

```
Log-odds matrix for Motif   0 rGAA..TtctrGAA (0)
#        0         1         2         3         4         5         6         7         8         9        10        11        12        13   
#A     0.743    -1.052     1.647     1.443    -1.558    -0.374    -3.255    -5.001    -0.793    -2.480     1.175    -3.678     1.635     1.629 
#C    -1.105   -10.336    -8.324    -3.641     0.691     0.311    -1.463    -0.208     1.931    -1.053    -2.000   -10.641    -2.819    -4.350 
#T    -3.868    -3.114    -4.032    -2.297     0.288    -0.426     1.428     1.320    -2.576     1.393    -5.066    -3.566    -5.030    -3.764 
#G     0.967     2.100    -4.305    -1.267     0.140     0.632    -1.563    -1.879    -2.088    -2.285     0.357     2.321    -8.368    -4.399 
Sequence Logo
#  --------------
#                 -- 2.30 bits
#             G  
#             G  
#   GA        GAA
#   GAA  T C  GAA
#   GAA  TTCT GAA
#  AGAA  TTCTAGAA
#  GGAA  TTCTAGAA -- 0.29 bits
#  --------------
#  rGAA..TtctrGAA
Source:  HSF1
Motif   0 rGAA..TtctrGAA (0)        (Bits: 14.14  MAP:  0.00   D: 0.000  -1) E:  0.000  ch:  0.00
AAAAAACCAAAGAA 0 2.66564019036
AAAACACCACAGAA 0 6.34181478692
AGAACAGCCCAGAA 0 12.1162311612
AGAACCGCCGAGAA 0 11.5694520088
AGAACCTCCGAGAA 0 14.5611965064
AGAACCTGCTAGAA 0 16.5680637894
AGAACCTTCTAGAA 0 19.7669118865
AGAACCTTCTAGAA 0 19.7669118865
CGAACCTTCTAGAA 0 17.9190181888
CGAAGGTTCTAGAA 0 17.6896096981
GGAAGGTTCTAGAA 0 19.761489259
GGAAGGTTCTAGAA 0 19.761489259
GGAAGGTTCTCGAA 0 16.5861453685
GGAAGGTTCTGGAA 0 18.9435955025
GGAAGGTTCTGGAA 0 18.9435955025
GGAATGTTCTGGAA 0 19.0918355924
GGACTGTTCTGGAA 0 14.0084990288
GGAGTTTTCTGGAA 0 15.32449778
GGAGTTTTCTGGAA 0 15.32449778
GGATTTTTGTGGCC 0 -0.158188860768
**************
MAP Score: 0.000000
```
